# Characterization and application of synthesized calcium alginate-graphene oxide for the removal of Cr^3+^, Cu^2+^ and Cd^2+^ ions from tannery effluents

**DOI:** 10.1016/j.clwat.2024.100016

**Published:** 2024-06

**Authors:** Sobur Ahmed, Tasrina Rabia Choudhury, Md. Zahangir Alam, Mohammad Nurnabi

**Affiliations:** a Institute of Leather Engineering and Technology, University of Dhaka, Hazaribagh, Dhaka 1209, Bangladesh; b Analytical Chemistry Laboratory, Atomic Energy Centre, Atomic Energy Commission, Dhaka, Bangladesh; c Department of Applied Chemistry and Chemical Engineering, University of Dhaka, Dhaka 1000, Bangladesh

**Keywords:** Adsorbent, Calcium alginate, Graphene oxide, Tannery effluent

## Abstract

Environmental sustainability has gained acceptance to achieving the goal of a secure ecosystem with a reliable management system. Heavy metal remediation of aqueous streams is of special concern due to the intractability and persistence in the environment. Adsorption is a potential alternative to the existing inefficient conventional technologies for the removal and recovery of metal ions from aqueous solutions and becomes vital to align with the Sustainable Development Goals (SDGs) and mitigate the adverse environmental and social impacts. Calcium Alginate-Graphene oxide (CA-GO) composite has been synthesized for the adsorption of heavy metals including Cr^3+^, Cu^2+^, and Cd^2+^ ions from tannery effluents. Graphene oxide is prepared from commercial graphite powder and reacted with sodium alginate and calcium chloride to form the beads of CA-GO composite. The developed composite was characterized by FTIR, elemental analysis, SEM, XRD analysis, and Raman spectroscopy. Moreover, the effect of pH, adsorbent dosage, contact time, and initial concentration of metal ions on the adsorption capacity were investigated through batch experiments. At a pH>3.0 (pHzpc), the carboxyl group of CA-GO was deprotonated to make the surface negatively charged and facilitate metal adsorption. The optimum pH and maximum adsorption capacity of CA-GO for removal of Cr(III), Cu(II), and Cd(II) were 4.5, 6.0, and 7.0, and 90.58, 108.57, and 134.77 mg g^−1^, respectively. The kinetics, adsorption isotherms, and thermodynamics were studied to determine the adsorption mechanism. The kinetic of adsorption adopted the second-order model. Thermodynamic parameter were calculated and the adsorption process was determined to be exothermic and spontaneous at room temperature. The developed composite has been efficaciously applied for the removal of metal ions and pollution from real tannery effluents.

## 1 Introduction

Leather production from animal hides/skins has been one of the oldest manufacturing methods since the beginning of human civilization to meet the demand for garments and foot covering (Appiah-Brempong et al., 2020, Mahdi et al., 2021). According to Sundar et al. (2011), the fabulous fibre texture of leather is still unmatched by any other manmade fabrics and other polymeric materials (Sundar et al., 2011). Moreover, the premium quality of leather and its associated products such as shoes, leather garments, gloves, etc. are unique, very comfortable, and hygienic to use as daily commodities (Shahriar et al., 2019). As a result, the leather industry has always been an important business sector as well as a source of employment for many countries (Sundar et al., 2011, Li et al., 2019). Despite having a substantial economic contribution, the leather industry is recognized as a potential pollutant-generating industry (Aktar et al., 2023, Hossain et al., 2023). In the process per ton of wet salted hides/skins, 500 kg chemicals, 15–50 m^3^ water, and 2600–11700 kW energy are consumed yielding only 200–250 kg finished leather remaining 72 kg chemicals inside and release 600–750 kg of various solid wastes (both tanned and untanned) and 30–35 m^3^ effluents (Li et al., 2019, Mozhiarasi et al., 2021, Paul et al., 2013). These effluents have no further possible application and need proper treatment before discharging them into the environment, as they contain a major quantity of heavy metals, such as Cr, Cu, Cd, Fe, Pb, etc., colorants, and other organic and inorganic pollutants that create toxicological problems for both environment and living species and decrease the quality of water as well (Hassen and Woldeamanuale, 2017, Meneberu, 2021). As a major pollutants toxicity of heavy metals is becoming an issue for ecological, nutritional, and environmental causes. Any metallic elements with high density, which is toxic (non-biodegradable) and poisonous even at low concentration is referred to as “heavy metals” (Nagajyoti et al., 2010). Tannery effluents contain a various toxic heavy metals, including Cr, Cd, Co, Pb, Ni, Se, and As (Lofrano et al., 2014). Scientists have reported that the disposal of untreated chromium enriched tannery effluents discharged from chrome-tanning and re-chroming operations deposit a notable amount of chromium complexes into water streams which causes river pollution in many parts of the world (de Aquim et al., 2019, Guo et al., 2021, Uddin and Jeong, 2021). Ninety percent of the leather industries use chromium as ‘Basic chromium sulfate’ [Cr(OH)(SO_4_)], for tanning of leather as they show exceptional shrinkage temperature and few distinctive properties (smooth grain, good elasticity, and resistance to atmospheric influences) (Gao et al., 2020). Previous studies showed that the pickled pelts uptake only 60 % of the employed chromium salts and the residual 40 % of them remained unreacted and completely discharged with wastewater, ranging between 2656 and 5420 mg/L that is much beyond its threshold limit in both surface water (0.1 mg/L) and industrial effluents (5 mg/L) (Ahmed et al., 2017, Hashem et al., 2020, Khalid et al., 2018, Mondal and Chakraborty, 2020). A substantial quantity of heavy metals, for example chromium, cadmium, copper, iron, lead, barium, etc. are discharged through wastewater during leather production (Das et al., 2010). These metal ions are first deposited into soil from leachates and become stable after reacting with organic soil matters/inorganic anions (nitrates and phosphates) and turns into less mobilized species (Xu et al., 2020). These species are highly biocompatible and stored into vegetative or reproductive parts of different plant species through bioaccumulation, then can affect human health and even causing mortality during long-term intake of such plants as a daily diet (Achmad and Auerkari, 2017, Chen et al., 2014, Olowoyo, 2023). In the previous study, author investigated the metal contents, such as chromium and cadmium in tannery effluents contaminated soil and found Cr = 100 mg/kg Dw and Cd = 1.5 mg/kg Dw (Ahmed et al., 2022), which exceeded the permissible limits recommended by DoE (2015). Therefore, proper treatment of chromium and other heavy metals containing tannery effluents should be of paramount interest to protect the environment and sustainability of this age-old industry (Alam et al., 2019, Al-Mizan et al., 2020). At present, several conventional and advanced wastewater treatment techniques (WWTTs) have been subjected to employ for remediation of toxic metals, proteinous particulates, refractory wastages (dyes and pigments), and other persistent organic pollutants (phthalates, polyphenols, etc.) from tannery effluents (Nur-E-Alam et al., 2020). The methods like ion exchange, chemical oxidation and reduction, membrane filtration, chemical precipitation, coagulation-flocculation, adsorption, biological treatment, electrochemical methods, etc. were developed for the removal of heavy metals from wastewater. A majority of those methods required sophisticated equipment and selective components (bacterial/fungal strains, chemical agents), which increase both machine maintenance and effluent treatment expenses, resulting in a promotion of the manifestation of economical and highly effective treatment technologies for the remediation of industrial effluents (Crini and Lichtfouse, 2019). Adsorption is considered the most capable of all WWTTs owing to its operational simplicity, inexpensiveness, noticeable removal capacity of wide-ranged chemicals from a very dilute aqueous solution, and less secondary waste formation (İnce and Kaplan İnce, 2017).

In recent times, graphene and graphene oxide (GO), a novel type of carbon materials has drawn massive interest of research for the treatment of wastewater as they exhibit huge adsorption capability towards different adsorbates. Heavy metals like Cd^+2^, Co^+2^, and Pb^+2^ from wastewater can be eliminated by using GO (Elgengehi et al., 2019, Zhao et al., 2011a, Zhao et al., 2011b). The activated crosslinked calcium alginate–graphene oxide beads were investigated for the removal of methylene blue dye and the pharmaceuticals famotidine and diclofenac with a range of physicochemical properties (Gunes et al., 2021). 3D calcium alginate/graphene oxide (3D CA/GO) was used to adsorb lead ion from aqueous solution (Wang et al., 2023). However, the GO is expensive and has some limitations in application and biotoxicity to human cells. It has also a tendency to aggregate in an aqueous solution resulting in a reduced surface area, which decreases the adsorption capacity. Hence, developing eco-friendly graphene-based adsorbents, such as porous calcium alginate-graphene oxide (CA-GO) composite with a stable structure and morphology can be a solution to these problems and novel ideas to apply. This sort of work has not been done yet. This research focuses on the preparation of graphene-based CA-GO hydrogel beads and finds out the application and effectiveness of remove and recover chromium, copper and cadmium from tannery effluent. The specific objectives included-

• Synthesis and characterization of calcium alginate-GO hydrogel beads in terms of various spectrophotometric and physicochemical analyses.
• Evaluation of the metal adsorption efficiency of calcium alginate-GO beads.
• Study the sorption kinetics, mechanism and thermodynamic effect on adsorption.
• Examination of compatibility of the prepared adsorbent on metal removal and its regeneration and reuse capability.
• Application of adsorbent in real tannery effluent sample to remove heavy metals.

## 2 Experimental

### 2.1 Chemicals

Chromium sulfate (Cr_2_(SO_4_)_3_.6H_2_O), copper chloride (CuCl_2_.2H_2_O) and cadmium sulfate (3CdSO_4_.8H_2_O) were collected from Merck, India and used to prepare the standard solution of Cr(III), Cu(II) and Cd(II). The chemicals were purchased from different sources, such as H_2_SO_4_ (98 %), and HNO_3_ (65 %) from Active Fine Chemicals (Bangladesh), NaNO_3_ from Unichem (China), graphite powder (99.5 %), KMnO_4_ (97 %), and H_2_O_2_ (30 %) from Merck (India), HCl from RCI Labscan (Thailand). Sodium alginate (Merck, India) and calcium chloride (Unichem, China) were procured and used for the preparation of graphene oxide, calcium alginate (CA) and calcium alginate-graphene oxide (CA-GO) composite.

### 2.2 Instruments

In this study, the HANNA Instrument (HI-98107, Germany) was used to judge the pH and a mercury digital thermometer was used to measure the temperature. TDS, EC and NaCl (%) of effluent were investigated with the HANNA instrument (HI-2300). The BOD_5_ and COD of both treated and untreated water were evaluated using (APHA-5220C, 1997) and (APHA-5210 B, 2001) methods. COD was determined by following the official standard method (DIN 38409). The heavy metals (such as Cr, Cu, and Cd) in tannery effluent, were analyzed after acid digestion using inductively coupled plasma-mass spectrometry (Agilent 7900 ICP-MS, Model no. G8403A, Agilent Technologies International Japan Ltd.). To characterize the calcium alginate graphene oxide (CA-GO) composite, Fourier Transform Infrared Spectroscopy (FTIR) (8400S Shimadzu, Japan), Scanning Electron Microscopy (SEM) (JEOL, USA), X-ray diffraction (XRD) analysis, and Raman spectroscopy were used. Malvern Zetasizer (Nano-ZS ZEN 3600) was used to measure the zeta potential for the produced adsorbent as a function of pH. The elemental analysis of GO was conducted using an X-ray photoelectron spectrometer (Model: K-ALPHA, Thermo Fisher Scientific, Czech Republic). Using a BET sorptometer (Model: BET-201-A, PMI, USA), the Brunauer-Emmett-Teller (BET) surface area, pore volume, and pore size distribution of CA-GO were examined.

### 2.3 Methods

#### 2.3.1 Preparation of graphene oxide (GO)

The modified Hummer's approach, as described in the earlier work (Huber et al., 2021), was used to prepare GO. To make a thick paste, 3.0 g of graphite powder, 9 g of KMnO_4_, 1.5 g of NaNO_3_, and 75 mL of concentrated H_2_SO_4_ and HNO_3_ (3:1) were added over the course of two hours while being rapidly mixed. The mixture was then left at room temperature for the entire night. Then the mixture was diluted with 120 mL of deionized (DI) water and agitated for 4 h at 35 °C in an oil bath to achieve a deep brown mixture. To obtain GO as a suspension, more stirring was done while adding 5 % HCl (200 mL), 30 % H_2_O_2_ (20 mL), and DI water (420 mL). The suspension was washed with water many times before being centrifuged until it reached neutrality.

#### 2.3.2 Synthesis of CA beads

Calcium-alginate beads were synthesized using a calcium chloride solution and a sodium alginate solution. 2 g of sodium alginate were first dissolved in 50 mL of DI water while being stirred magnetically at 150 rpm for three hours at room temperature. The mixture was then ultra-sonicated for thirty minutes to create a homogenous dispersion in the solution media. Following that, this dispersion was cautiously added to an aqueous coagulation bath that contained 6 % CaCl_2_ (w/v). To stop the beads from clumping together, the bath was constantly stirred with a magnetic stirrer. To fully finish the process of cross-linking and the creation of calcium alginate beads, the beads were left undisturbed for a full day.

#### 2.3.3 Synthesis of CA-GO beads

**Fig. 1:**
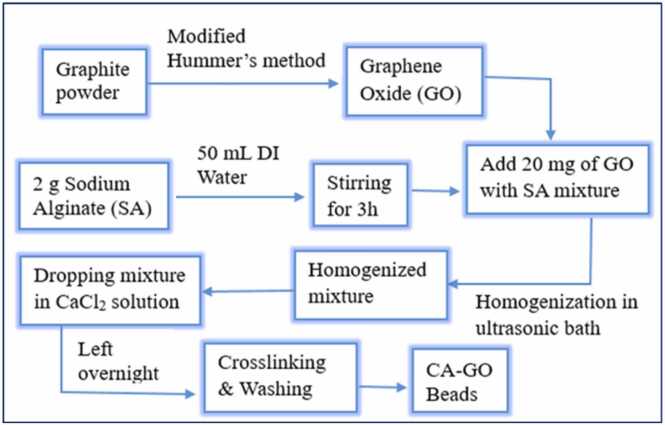
Schematic flow chart for preparation of CA-GO beads.

**Fig. 2:**
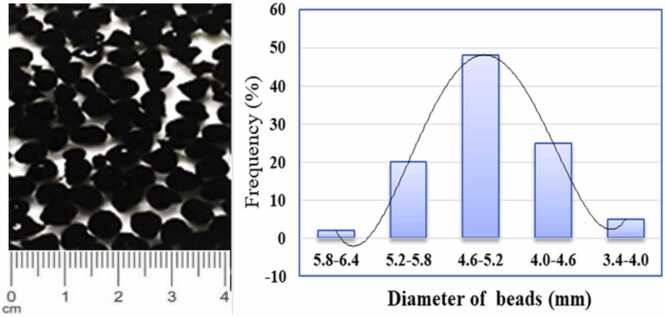
Image of prepared CA-GO composite beads and their size distribution histogram.

To prepare CA-GO beads, the first commercial graphite powder was oxidized to graphene oxide using modified Hummer’s method. In 50 mL of DI water, 2 g of sodium alginate was dissolved under magnetic stirring at 150 rpm for 3 h at room temperature. Then the addition of 20 mg GO was followed by homogenization using an ultra-sonic device. This mixture was then carefully dropped into an aqueous coagulation bath containing 6 % CaCl_2_ w/v solution. The bath was continuously agitated with a magnetic stirrer to prevent agglomeration of CA-GO beads. The beads were left for 24 h without agitation to complete cross-linking. Fig. 1 is a schematic flow chart for the preparation of CA-GO. Fig. 2 shows the image of prepared CA-GO composite beads and their size distribution histogram (average diameter 4.8 mm in wet condition).

##### 2.3.3.1 Structure of CA-GO beads

**Fig. 3:**
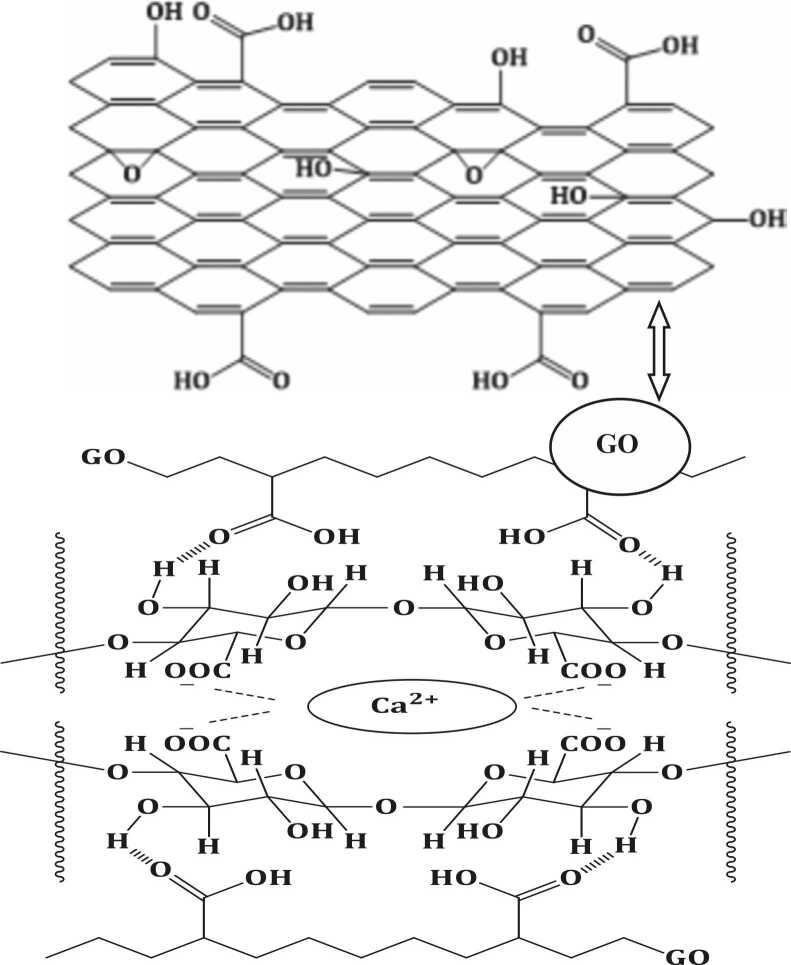
Structure of CA-GO composite beads.

Fig. 3 shows the structure of CA-GO composite beads where the graphene sheet is mentioned separately. The sodium cations in the alginate are driven away from the -COONa on the mannuronic and guluronic acid residues in the presence of the CaCl_2_ solution resulting in crosslinking of the carboxylate ions with the Ca^2+^ cations forming calcium alginate, (C_12_H_14_CaO_12_)n.

#### 2.3.4 Parameter exploration and data treatment

Several batch studies were run at a range of pH, adsorbent dosages, metal concentrations, and times to decide the ability of adsorption. Amounts of metals in the untreated and treated water samples were determined using coupled plasma-mass spectrometry (ICP-MS). To determine the capacity of adsorbent for metal adsorption, a fixed quantity of adsorbent was introduced to metal solutions of a specific concentration and pH. After filtering and stirring the mixtures, the ICP-MS was utilized to measure the concentration change. (1), (2) were used to determine the adsorption capacity, q_t_ (mg/g) at time t and the percentage of metal removal, respectively. Eq. (3) provided the adsorption capacity at equilibrium, q_e_ (mg/g).

(1) $$\mathrm{Adsorption}\;\mathrm{capacity}\;\mathrm{at}\;\mathrm{time}\;t,q_{t}=\frac{\left(C_{0}-C_{t}\right)\times V}{W}$$

(2) $$\%\;\mathrm{of}\;\mathrm{removal}=\frac{\left(C_{0}-C_{e}\right)}{C_{0}}\times 100$$

(3) $$\mathrm{Adsorption}\;\mathrm{capacity}\;\mathrm{at}\;\mathrm{equilibrium},q_{e}=\frac{\left(C_{0}-C_{e}\right)\times V}{W}$$

Where, C_0_, C_t_ and C_e_ are the concentration of metal ions at initial, concentration of at time t and concentration at equilibrium (mg/L), respectively, V refers to the volume of metal ion containing solution (L), and W indicates the mass of adsorbent employed for adsorption (g). The isotherms for the Langmuir Eq. (4) and Freundlich Eq. (6) were used to explain the equilibrium of the adsorption process.

(4) $$\frac{C_{e}}{q_{e}}=\frac{1}{q_{m}\text{b}}+\frac{1}{q_{m}}C_{e}$$

(5) $$R_{L}=\frac{1}{1+C_{m}b}$$

(6) $$\ln q_{e}=\ln K_{F}+\frac{1}{n}\ln C_{e}$$

Where b is Langmuir constant (L/mg), C _e_ is equilibrium metal ion concentration (mg/L), and q_e_ and q_m_ are equilibrium adsorption capacity and theoretical maximum adsorption capacity (mg/g), respectively. By plotting the C_e_/q_e_ vs. C_e_ value, the theoretical maximum adsorption capacity q_m_ was estimated following the Langmuir model. The separation factor, R_L_ value was calculated by the Eq. (5), where C_m_ is the maximum concentration of initial metal employed to assess the favorability of adsorption.

The pseudo-first-order (PFO) and pseudo-second-order (PSO) models were assessed using (7), (8), respectively, to evaluate the kinetics of the adsorption process.

(7) $$\log\left(q_{e}-q_{t}\right)=\log q_{e}-\left(\frac{k_{1}}{2.303}\right)t$$

(8) $$\frac{t}{q_{t}}=\left(\frac{1}{k_{2}q_{e}²}\right)+\left(\frac{1}{q_{e}}\right)t$$

Where k_1_ and k_2_ are the rate constants (g/mg min), q_e_ indicates adsorption at equilibrium (mg/g), and q_t_ is adsorption capacity at any time t (mg/g).

The (9), (10) were used to calculate the Gibbs free energy change (ΔG) in thermodynamic analysis.

(9) $$∆G=-\mathrm{RT}\ln k_{d}$$

Where T is the absolute temperature (K), k_d_ is the distribution coefficient for the equilibrium sorption, and R is the universal gas constant (8.314 J mole^−1^ K^−1^).

(10) $$k_{d}=\frac{q_{e}}{C_{e}}$$

The change in entropy (ΔS) and enthalpy (ΔH) was calculated using the linearized van't Hoff isotherm Eq. (11).

(11) $$\ln k_{d}=\frac{-∆H}{\mathrm{RT}}+\frac{∆S}{R}$$

## 3 Results and discussion

### 3.1 Characterization

The CA-GO was characterized by different instrumental techniques. FTIR was used to identify the functional groups, while SEM, XRD, Raman Spectrum, and BET analysis were used to examine the morphology, surface structure, chemical characteristics, and crystalline features.

#### 3.1.1 Spectral analysis

**Fig. 4:**
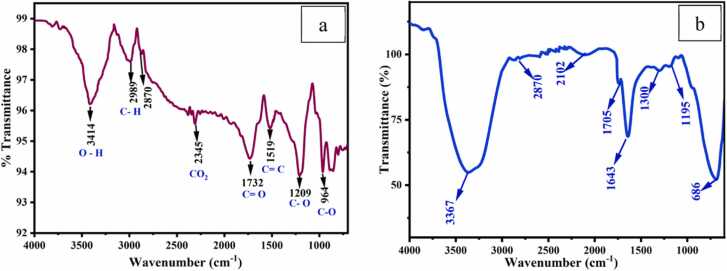
FTIR of (a) GO and (b) CA-GO.

In the infrared region, quite significant adsorptive peaks were observed in the GO and CA-GO spectrum at various wavenumbers (Fig. 4). The O-H stretching, C-H stretching, C=O stretching vibration of carboxylic group, and C-O stretching vibration of C-O-C group were correlated with the peaks identified at 3414, 2989, 1732, and 1209 cm^−1^. Prior to adsorption, the CA-GO spectra showed peaks at 3367, 2870, 1726, and 1099 cm^−1^, which corresponded to the stretching vibrations of the carboxylic group, the O-H stretching, the C-H stretching, and the C-O stretching vibration in the C-O-C group. The peaks of O-H stretching vibration for GO shifted from 3414 to 3367 cm^−1^, C=O shifted from 1732 to 1726 cm^−1^, and C-O shifted from 1209 to 1099 cm^−1^ during the production of CA-GO beads. The developing composition of the CA-GO beads caused some minor changes to several other peaks as well.

#### 3.1.2 Microscopic analysis

**Fig. 5:**
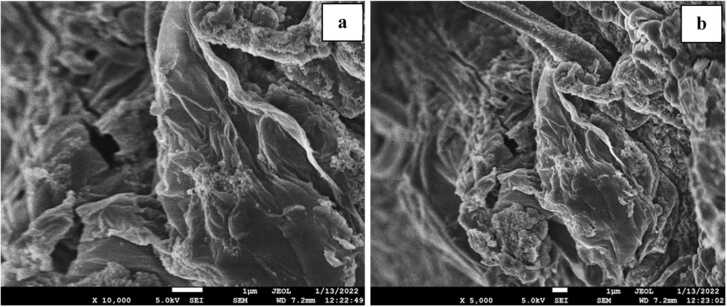
SEM image of CA-GO composite beads a) 10,000 × and b) 5000 × magnification.

The SEM micrograph of the CA-GO image was captured at 10,000× and 5000× magnification and 7.2 mm working distance at vacuum mode with 5.00 kV. The morphological structure of the beads was observed with increased porosity and roughness because of the incorporation of GO in the sodium alginate which finally reacted with CaCl_2_ (Fig. 5). The beads had a greater surface available for interaction between adsorbate and adsorbent.

#### 3.1.3 XRD analysis

**Fig. 6:**
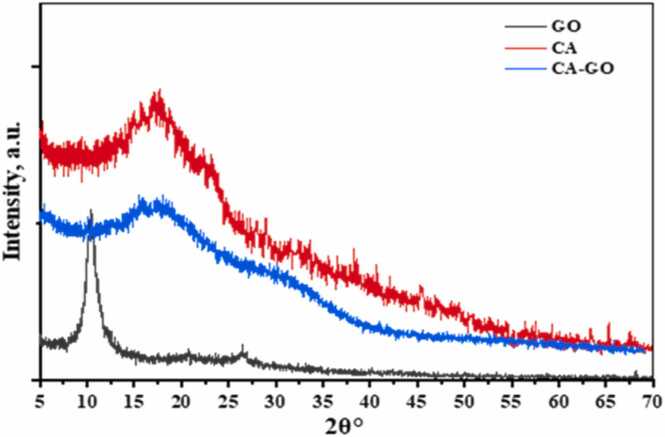
XRD patterns of CA-GO composite beads.

The XRD patterns of GO, CA and CA-GO composite are represented in Fig. 6. The XRD pattern of GO represented peaks at 2θ = 10.399° corresponding to an interlayer spacing of 8.58 Å. The value of 2θ was 17.76° corresponding to interlayer spacing 3.341 Å indicating an amorphous structure. The XRD pattern was observed at 2θ = 17.5° and interlayer spacing of 3.023 Å for CA-GO. These results revealed that the diffraction as well as interlayer spacing of CA-GO becomes close to calcium alginate, which were amorphous.

#### 3.1.4 Raman spectrum analysis

**Fig. 7:**
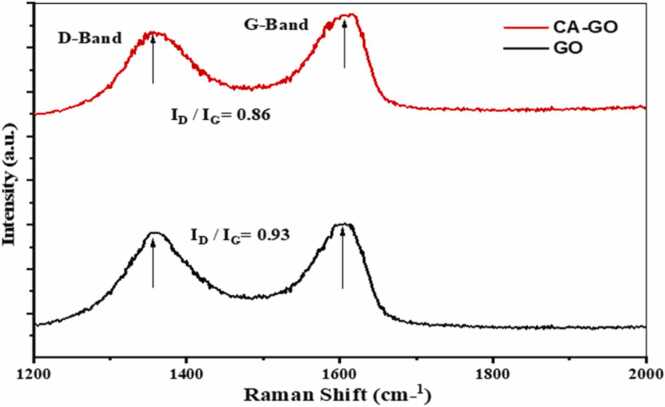
Raman Spectrum of GO and CA-GO composite beads.

The presence of the D-band and G-band was confirmed by the Raman spectrum of GO and CA-GO (Fig. 7). The D-band indicate the existence of defect sites on the adsorbents. The values of D-band and G-band for GO are 1356 and 1607 cm^−1^ whereas these values for CA-GO were 1359 and 1670 cm^−1^, respectively. The structural disorder is measured by the integrated intensity ratio of D-band and G-band (I_D_/I_G_), which are 0.93 for GO and 0.86 for CA-GO, thus CA-GO has fewer defective sites to GO.

#### 3.1.5 Brunaur-Emmett-Teller (BET) analysis

**Fig. 8:**
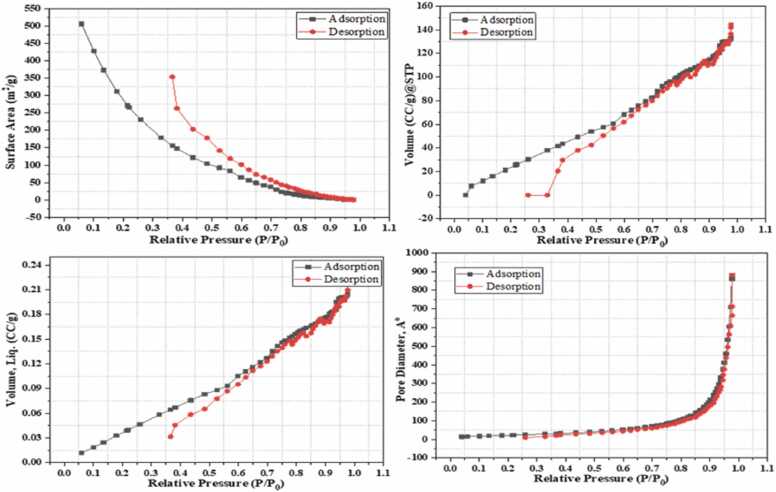
BET analysis of CA-GO composite beads.

A nitrogen sorption system was used to analyze the surface area and pore diameter of CA-GO (Fig. 8). The total pore volume and specific surface area of spongy CA-GO were observed 0.2221 cc/g and 188.27 m^2^ g^−1^, respectively (Table 1). The average pore diameter, as determined by the Barrett-Joiner-Halenda (BJH) method, was 47.18 Å, suggesting that CA-GO is composed of mesopores (Rafatullah et al., 2009). The CA-GO total pore volume was determined to be 0.2221 cc/g.

**Table 1:** Parameters of BET analysis for CA-GO composite beads.

BET Parameter	Results
BET-specific surface area	188.27 m^2^ g^−1^
Total pore volume	0.2221 cc/g
Average pore diameter	47.18 Å

#### 3.1.6 Zeta potential charge and ionic behavior of CA-GO beads

**Fig. 9:**
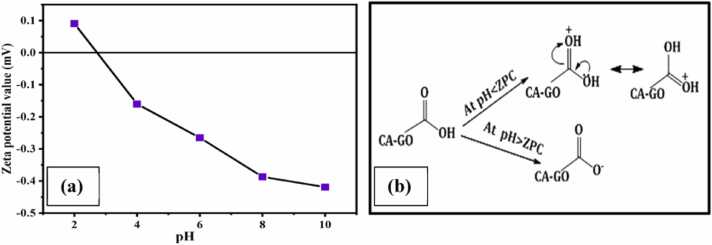
(a) Zeta potential value (b) ionic behavior of CA-GO beads at different pH.

The pH of CA-GO composite dispersion was adjusted to 2.0, 4.0, 6.0, 8.0, and 10.0 using dilute HCl and NaOH solution. The zeta potential charge (ZPC) was evaluated and it found that the zeta potential value of CA-GO was positive (0.0908 mV) at pH 2.0, which became negative (-0.160 to −0.419 mV) due to a pH increase from 4.0 to 10.0. The ZPC of CA-GO was zero at a pH of nearly 3.0 (Fig. 9. (a)). The pH at higher than the ZPC, carboxyl group of CA-GO was deprotonated and negatively charged and vice versa (Fig. 9. (b)).

Therefore, the metal adsorption capacity was favored at higher pH due to the electrostatic interaction between the anionic surface charge of CA-GO and cationic metal ion.

Furthermore, there was strong competition between metal ions and H^+^ at lower pH levels, where protons occupied the majority of the adsorbent surface due to their smaller size. At lower pH values, both of these situations resulted in reduced metal ion adsorption (Mohubedu et al., 2018, Diagboya and Dikio, 2018a). However, at pH over the ZPC, the deprotonated negatively charged carboxylic group of CA-GO led to an electrostatic attraction between the adsorbent surface and metal ions (Sera et al., 2021). Moreover, a higher pH resulted in a lower proton concentration, which diminished proton competition with cations and increased metal ion adsorption (Diagboya and Dikio, 2018b).

### 3.2 Process factors and their influence on adsorption

#### 3.2.1 Effects of pH

**Fig. 10:**
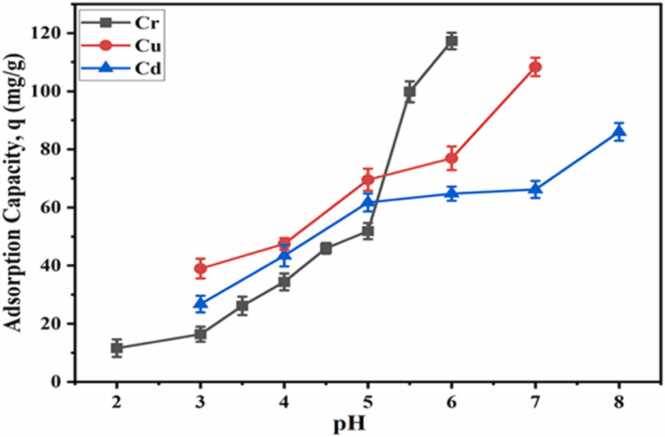
Effect of pH on adsorption capacity of CA-GO for Cr(III), Cu(II), and Cd(II).

The adsorption capacity of CA-GO is substantially influenced by pH. The impact of pH was investigated using Cr(III) salt solution (20 mL, 146.7 ppm), Cu(II) salt solution (20 mL, 124.3 ppm) and Cd(II) salt solution (102.69 ppm, 20 mL) treated with CA-GO beads (1.116 g/L, 22.0 mg), respectively at the pH range 2.0–6.0 for Cr(III), 3.0–7.0 for Cu(II), and 3.0–8.0 for Cd(II). They were agitated at room temperature in an orbital shaker at rpm 150 for 2 h. After the mixtures were filtered, AAS calculated the concentration changes. The outcome showed that the adsorption capacity increased at elevated pH (Fig. 10). However, after reaching pH 5.0 the removal rate of Cr was drastically increased (51.88 mg/g to 99.82 mg/g) owing to chromium precipitation. Hence, the optimum pH for chromium was chosen as 4.5, at which the adsorption capacity and % removal were 45.96 % and 34.97 mg/g. Similarly, Cu(II) adsorption capacity increased dramatically at pH 7.0 and reached maximum of 108.37 mg/g due to precipitation (Fig. 10) as pH higher than 6.0, the copper is precipitated. Therefore, pH 6.0 was determined to be optimum, where the percentage of Cu(II) removal and the adsorption capacity of CA-GO were found to be 85.58 % and 76.95 mg/g, respectively. Furthermore, Cd(II) precipitation was detected at pH>7.0 during Cd(II) adsorption on CA-GO. Thus, pH 7.0 was determined to be optimum, at which Cd(II) removal percentage and adsorption capacity were 71.95 % and 66.21 mg/g, respectively.

#### 3.2.2 Effect of dosage

**Fig. 11:**
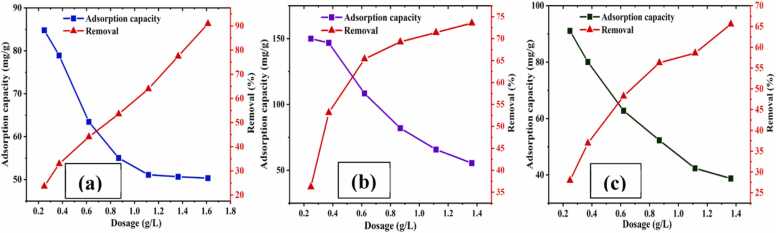
Effect of dosage on adsorption capacity and % of removal of (a) Cr(III), (b) Cu(II), and (c) Cd(II).

Using 89.23 ppm Cr(III) solution at pH 4.5, the effect of CA-GO dosage on Cr(III) adsorption was examined. The procedure was carried out using the various dosages (0.248–1.612 g/L) for two hours at 150 rpm. It was shown that with the raising of dosage, the percentage of Cr(III) elimination rose, although the adsorption capacity was lowered (Fig. 11. (a)). The optimum dosage was chosen as 0.62 g/L based on experimental results and this was kept constant throughout the investigation. Comparably, using a standard copper salt solution (102.8 ppm, 20 mL) at optimal pH (6.0) with varying dosages (0.248–1.364 g/L), the impact of CA-GO dosage on Cu(II) adsorption was investigated. Here, the dosage (0.6 g/L) demonstrated the optimum result since it met the balance of the adsorption capacity and percentage of removal (Fig. 11. (b)).

Given optimization of adsorbent dosage, standard cadmium salt solution (20 mL, 80.68 ppm) was treated with different dosages (0.248–1.364 g/L) at optimum pH (7.0). The adsorbent capacity was decreased with increasing adsorbent dosage, however, per cent of cadmium removal was increased (Fig. 11. (c)). The unsaturation of some active sites of adsorbent (CA-GO) and few of them remained unreacted at the higher dosage of adsorbent. The adsorbate quantity per unit mass of adsorbent dropped when the adsorbent dosage was increased (Alka Shukla et al., 2002). The optimum dosage was determined to be 0.6 g/L based on the experiment results, and this was kept constant for the duration of the investigation.

#### 3.2.3 Time and metal concentration effect

**Fig. 12:**
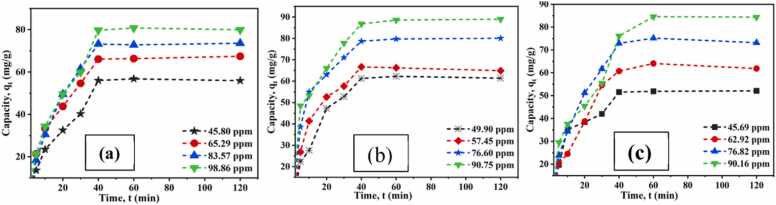
Effect of time and concentration on adsorption of (a) Cr(III), (b) Cu(II), and (c) Cd(II) on CA-GO at different concentrations.

Twenty milliliters of chromium salt solutions at different concentrations were prepared at pH 4.5 to examine the impact of Cr(III) concentration and contact time on adsorption capacity. Each solution was then treated with 0.62 g/L of CA-GO and agitated for 5–120 min. As time increased, the adsorption capacity of CA-GO increased until it reached equilibrium and then became constant after 40 min (Fig. 12. (a)). Due to a rise in the concentration gradient between metal ions in the bulk solution, which favoured mass transfer of chromium ions onto the adsorbent surface, equilibrium metal ion adsorption capacity increased with increasing initial metal ion concentration (Tajiki and Abdouss, 2017). However, the % removal was higher at a lower initial chromium concentration since the amount of Cr was less as compared to the amount of adsorbent, CA-GO composite. Similarly, copper salt solution (20 mL) of different concentrations (49.90, 57.45, 76.60, and 90.75 ppm) was used for a certain period (5–120 min) at pH (6.0) and dosage (0.6 g/L). After 40 min, the adsorption process reached equilibrium (Fig. 12. (b)).

Furthermore, standard cadmium salt solution (20 mL) of different concentrations (45.69, 62.92, 76.82, and 90.16 ppm) at pH (7.0) and dosage (0.6 g/L) were treated with batch experiments for a certain time (0–120 min). The adsorption process of cadmium by CA-GO also reached an equilibrium at around 40 min. The adsorption capacity of CA-GO improved while the percentage of Cd removal decreased as the starting concentration of Cd increased (Fig. 12. (c)).

#### 3.2.4 Adsorption isotherms

The distribution of ions containing chromium, copper, and cadmium on the CA-GO surface was examined using the Langmuir and Freundlich models. Langmuir isotherms provide assumption about monolayer adsorption and Freundlich isotherm gives assumptions about multilayer adsorption.

##### 3.2.4.1 Langmuir model

**Fig. 13:**
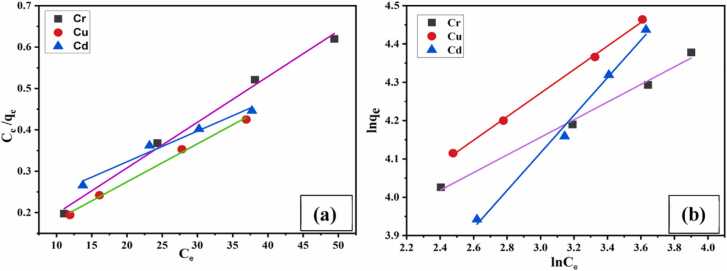
(a) Langmuir and (b) Freundlich isotherm for the adsorption of Cr(III), Cu(II), and Cd(II) on CA-GO.

By employing Eq. (4) to plot C_e_/q_e_ versus C_e_ value, the Langmuir model was confirmed. For the adsorption of Cr(III), Cu(II), and Cd(II), a linear connection with a satisfactory regression coefficient was found between C_e_/q_e_ and C_e_. The slope yielded the maximal theoretical adsorption capacity, q_m_, and Eq. (5) was used to get the separation factor, RL. The regression coefficient (R^2^), q_m_, b and R_L_ value were observed 0.993, 90.58 mg/g, 0.126 and 0.074 for Cr(III), 0.996, 108.57 mg/g, 0.102 and 0.097 for Cu(II), and 0.975, 134.77 mg/g, 0.0425 and 0.207 for Cd(II) adsorption, respectively, which indicated the favorable monolayer adsorption process of CA-GO (Table-2, Fig. 13. (a)).

##### 3.2.4.2 Freundlich model

The adsorption process on the heterogeneous surface is assumed to be multilayered by the Freundlich isotherm model. It also illustrates how numerous active centers with different energy levels are distributed exponentially for adsorption. The plot between lnC_e_ and lnq_e_ yielded the K_F_ (Freundlich constant) and n values, which are shown in Fig. 13. (b). A linear connection was found with excellent regression coefficient (R^2^) for Cr, Cu and Cd ions, which were 0.992, 0.998 and 0.992. The n value was calculated using the Eq. (6) and the results were 4.346, 3.254 and 2.037, indicating that the multilayer adsorption was of a moderate to good quality (Table 2).

**Table 2:** The values of q_m_, b, R_L_, n, k_F_ and R^2^ of adsorbent CA-GO for isotherms.

Parameters		q_m_, (mg g^−1^)	b (L mg^−1^)	R^2^	R_L_	n	k_F_
Langmuir isotherm	Cr(III)	90.58	0.126	0.993	0.074	-	-
	Cu(II)	108.57	0.102	0.996	0.097		
	Cd(II)	134.77	0.0425	0.975	0.207		
Freundlich isotherm	Cr(III)	-	-	0.992	-	4.346	32.01
	Cu(II)	-	-	0.998	-	3.254	28.49
	Cd(II)	-	-	0.992	-	2.037	14.06

The different parameters of both isotherms were enlisted in Table 2, which evidenced that both the models were followed by CA-GO for Cr(III), Cu(II) and Cd(II) adsorption.

#### 3.2.5 Adsorption kinetics

Adsorption kinetics was examined to evaluate an adsorbent's effectiveness and find more about the underlying mechanisms. An efficient metal removal procedure requires a quick response rate, a short adsorption time, and a high adsorption efficiency. These process-defining characteristics were made clear by using reaction rate equations to describe the change in the number of active sites on the material surface during the removal process (Sachin et al. 2023). Applying pseudo-first-order and second-order kinetic models assisted in describing the adsorption mechanisms of CA-GO.

##### 3.2.5.1 Pseudo-first-order (PFO) kinetics

**Fig. 14:**
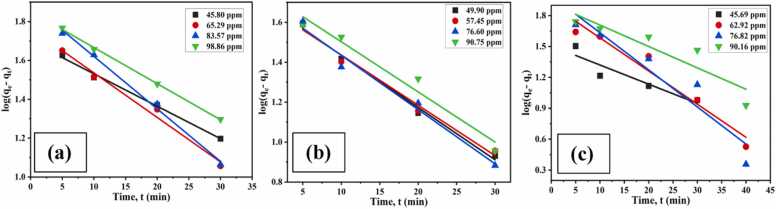
PFO kinetics of CA-GO for the adsorption of (a) Cr(III), (b) Cu(II), and (c) Cd(II).

A PFO kinetics was created by plotting log(q_e_-q_t_) versus t using the Eq. (7) at various initial concentrations of chromium, copper and cadmium, where linear relation between log(q_e_-q_t_) and t was observed (Fig. 14. (a), (b) (c)).

##### 3.2.5.2 Pseudo-second-order (PSO) kinetics

**Fig. 15:**
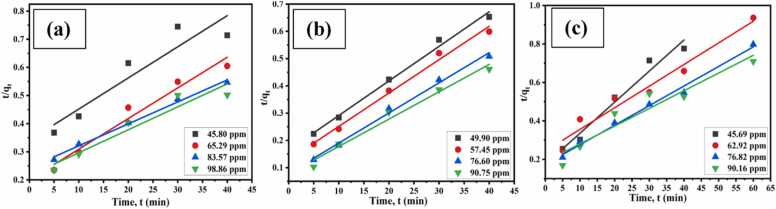
PSO kinetics of CA-GO for the adsorption of (a) Cr(III), (b) Cu(II), and (c) Cd(II).

A PSO kinetics was achieved by potting t/q_t_ against t using Eq. (8) and a linear relationship between t/q_t_ and t was observed (Fig. 15. (a), (b) (c)).

The adsorption capacity closely matched the experimental results, and it was found that the correlation coefficient (R^2^) values of PFO kinetics for chromium(III) adsorption on CA-GO were much greater than those of PSO kinetics (Table 3a, Table 3b, Table 3c). However, in case of copper(II) and cadmium(II) adsorption, the regression coefficient (R^2^) values of the PSO kinetics model and experimental values of q_e_ were noticeably higher than the PFO model. Nonetheless, the cadmium experimental results showed good agreement with the PFO kinetics. Thus, physicochemical adsorption was the mode of cadmium adsorption on CA-GO.

**Table 3a:** Kinetic parameters for the adsorption of Cr(III).

Kinetics model	Parameters	Initial concentration of Cr(III)			
		45.80 ppm	65.29 ppm	83.57 ppm	98.86 ppm
Pseudo-first-order	q_e_* (mg g^−1^)	56.02	66.08	73.24	79.74
	k_1_ (1/min)	0.038	0.051	0.0621	0.0426
	R^2^	0.994	0.986	0.995	0.998
	q_e_^**^ (mg g^−1^)	49.79	58.07	77.62	71.12
Pseudo-second-order	k_2_ (g/mg min)	3.548 × 10^−4^	5.823 × 10^- 4^	3.048 × 10^- 4^	2.511 × 10^- 4^
	R^2^	0.875	0.965	0.926	0.993
	q_e_^**^ (mg g^-1^)	90.91	92.59	123.45	128.04

**Table 3b:** Kinetic parameters for the adsorption of Cu(II).

Kinetics model	Parameters	Initial concentration of Cu(II)			
		49.90 ppm	57.45 ppm	76.60 ppm	90.75 ppm
Pseudo-first-order	q_e_* (mg g^-1^)	61.27	66.69	78.71	86.80
	k_1_ (1/min)	0.0645	0.0575	0.0621	0.0575
	R^2^	0.991	0.983	0.979	0.963
	q_e_^**^ (mg g^-1^)	49.65	48.75	50.82	56.70
Pseudo-second-order	k_2_ (g/mg min)	5.99 × 10^- 4^	1.12 × 10^- 3^	1.52 × 10^- 3^	1.29 × 10^- 3^
	R^2^	0.992	0.990	0.993	0.980
	q_e_^**^ (mg g^-1^)	79.36	83.33	90.91	100.00

**Table 3c:** Kinetic parameters for the adsorption of Cd(II).

Kinetics model	Parameters	Initial concentration of Cd(II)			
		45.69 ppm	62.92 ppm	76.82 ppm	90.16 ppm
Pseudo-first-order	q_e_* (mg g^-1^)	51.53	64.08	75.15	84.56
	k_1_ (1/min)	0.0414	0.0736	0.0829	0.0476
	R^2^	0.863	0.954	0.908	0.837
	q_e_^**^ (mg g^-1^)	32.06	80.48	98.51	82.32
Pseudo-second-order	k_2_ (g/mg min)	1.479 × 10^- 3^	0.497 × 10^- 3^	0.575 × 10^- 3^	0.43 × 10^- 3^
	R^2^	0.969	0.961	0.991	0.913
	q_e_^**^ (mg g^-1^)	62.50	90.91	100.00	111.11

*Experimental; **Theoretical.

#### 3.2.6 Thermodynamic analysis

**Fig. 16:**
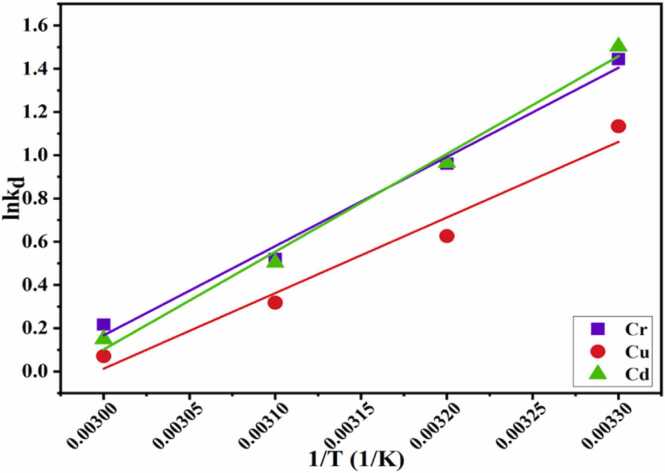
Plot for van’t Hoff equation for Cr(III),) Cu(II), and Cd(II) adsorption.

The thermodynamic analysis of an adsorption system determined its viability and randomness in terms of temperature. Changes in thermodynamic parameters for Cr^3+^, Cu^2+^ and Cd^2+^ adsorption on CA-GO at various temperatures (298–328 K) were examined using the Eq. (11), including Gibb's free energy (ΔG), enthalpy (ΔS), and entropy (ΔH). To measure the enthalpy change (ΔH) and entropy change (ΔS), respectively, the slope and intercept were utilized. A linear relationship for adsorption was found while plotting lnk_d_ versus 1/T values (Fig. 16).

Using a dosage of 0.62 g/L of CA-GO and a pH 4.5 solution containing 55.33 ppm Cr(III) for each experiment, Gibb's free energy change for chromium adsorption onto CA-GO at diverse temperatures was examined. The solutions (20 mL each) were shaken at 298, 308, 318, and 328 K for 40 min, which was the ideal duration. For chromium adsorption, the computed Gibb's free energy was found to be −3.580, −2.461, −1.374, and 0.592 kJ mol^-1^ at 298, 308, 318, and 328 K, respectively. The enthalpy change (ΔH) and entropy change (ΔS) were found to be −34.295 and −0.1014 kJ mol^-1^, respectively (Table 4). As the temperature increased from 298 to 328 K, the value of ΔG increased from −3.580 to −0.592 kJ mol^-1^. As a result, at lower temperatures, the chromium(III) adsorption on CA-GO was physical and spontaneous.

**Table 4:** Thermodynamic parameters.

Adsorbate	T(K)	ΔG (kJ mol^-1^)	ΔH (kJ mol^-1^)	ΔS (kJ K^-1^ mol^-1^)
Cr(III)	2 9 8	-3.580	-34.295	-0.1014
	3 0 8	-2.461		
	3 1 8	-1.374		
	3 2 8	-0.592		
	2 9 8	-2.809	-29.07	-0.0871
Cu(II)	3 0 8	-1.603		
	3 1 8	-0.841		
	3 2 8	-0.193		
	2 9 8	-3.723	-37.59	-0.1119
Cd(II)	3 0 8	-2.469		
	3 1 8	-1.328		
	3 2 8	-0.408		

For Cu(II) adsorption, the measured enthalpy change (ΔH) was −29.07 kJ mol^-1^ and the entropy change (ΔS) was −0.0871 kJ K^-1^ mol^-1^. As the temperature increased from 298 to 328 K, ΔG changed from −2.809 to −0.193 kJ mol-1 (Table 4). Cu(II) was thus spontaneously adsorbed on CA-GO at lower temperatures and the adsorption was physical.

The estimated results revealed that as the temperature raised, cadmium adsorption capacity of CA-GO was reduced due to release of adsorbate from adsorbent (CA-GO), which was assisted by higher kinetic energy at increased temperature. Gibb's free energies were determined to be −3.723, −2.469, −1.328, and −0.408 kJ mol^-1^ at 298, 308, 318, and 328 K, respectively. The values of entropy change (ΔS) and enthalpy change (ΔH) were determined to be −0.1119 kJ K^-1^ mol^-1^ and −37.59 kJ mol^-1^, respectively. When temperature was increased from 298 K to 328 K, the value of ΔG was also increased from −3.723 to −0.408 kJ mol^-1^ (Table 4). Therefore, at a lower temperature, the cadmium(II) adsorption on CA-GO beads was physicochemical and spontaneous.

#### 3.2.7 Plausible mechanism

**Fig. 17:**
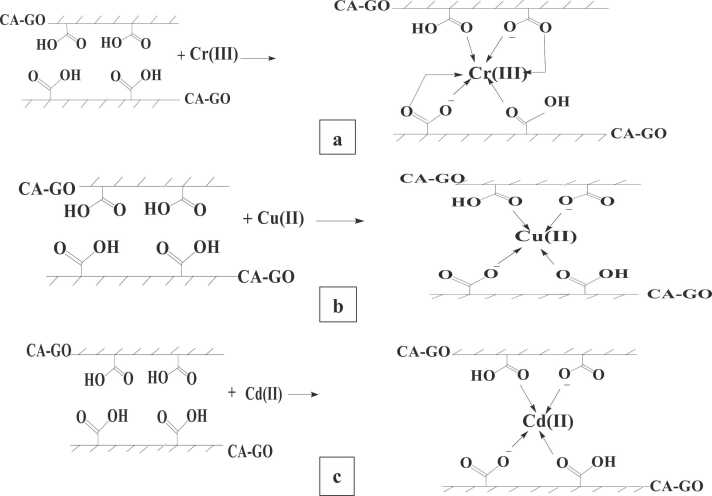
Possible adsorption mechanism of SA-GO for (a) Cr(III), (b) Cu(II) and (c) Cd(II).

The two most important factors have a key impact on adsorption of a solute on an adsorbent are the surface chemistry and pore density. Adsorption involves interactions between particles having opposite charges that produces a variety of bonding, comprising electrostatic bonds, hydrogen bonds, dipole-dipole interactions, van der Waals forces, and ion exchange. CA-GO exhibits electrostatic interaction with the cationic chromium ion due to its negative surface charge at pH 4.5. The carboxylate groups of CA-GO bind Cr(III) ions from the solution to create coordinate complexes (Fig. 17. (a)). Calcium alginate-graphene oxide surface possesses negative surface charge at pH higher than ZPC (pH 3.0) and unveils electrostatic interaction with cationic copper ion (Fig. 17. (b)). Electrostatic attraction, complexation and external ion exchange were mostly liable for adsorption of Cu(II) on CA-GO. The negatively charged carboxylate group of CA-GO at pH higher than its ZPC (3.0) bind cationic Cd(II) ions from the solution electrostatic interaction and forming coordinate complexes (Fig. 17. (c)).

#### 3.2.8 Regeneration studies

**Fig. 18:**
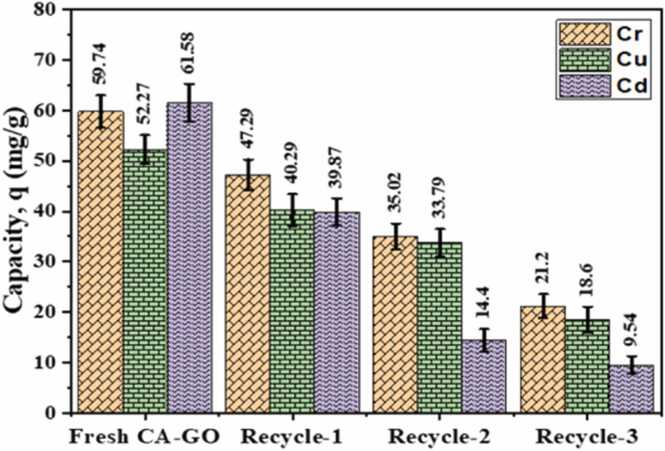
Regeneration of used CA-GO for Cr (III), Cu(II) and Cd(II) adsorption.

Regeneration studies of used adsorbents provide valuable information about commercial application of an adsorbent. 2.0 % HCl was used to regenerate CA-GO, which was then neutralized by repeatedly washing with distilled. The feasibility of reuse was investigated by drying the regenerated CA-GO and using it for further adsorption at optimum pH, duration and adsorption dosage. The experiments results showed that the adsorption capacity of regenerated CA-GO gradually decreased from 59.74 mg/g to 47.29, 35.02 and 21.20 mg/g for Cr(III), from 52.27 mg/g to 40.29, 33.79 and 18.45 mg/g for Cu(II) and from 61.58 mg/g to 39.87, 14.4 and 9.28 mg/g for Cd(II) after first, second and third recycle, respectively (Fig. 18).

#### 3.2.9 Application of CA-GO on tannery composite effluents

After evaluating ability of CA-GO to extract ions of chromium, copper, and cadmium from standard salt solutions, its efficacy in eliminating particular metal ions from actual tannery composite effluent was demonstrated. In order to examine the adsorption capabilities of CA-GO on tannery composite effluent, 500 mL of tannery effluent was mixed with 5.0 g of CA-GO adsorbent, and the mixture was agitated for 4 h at 150 rpm at room temperature. ICP-MS was used to determine the concentrations of Cr, Cu, and Cd both before and after adsorption. The results of the assessments of the other water quality parameters, including pH, TDS, EC, NaCl (%), BOD5, and COD, are shown in Table 5.

**Table 5:** Physicochemical features of composite tannery effluents before and after adsorption with CA-GO beads.

Parameters	Before adsorption	After adsorption	% of removal	DoE Standard
Cr(III) (ppm)	526.12	102.36	80.54	2.0
Adsorption capacity (mg/g)	-	42.38	-	
Cu(II) (ppm)	2.17	0.63	70.96	0.50
Cd(II) (ppm)	1.28	0.53	58.59	0.50
pH	5.25	5.80		6.0–9.0
TDS (ppm)	7,338	2698	63.23	2100
EC (µS/cm)	5,120	1,535	70.02	1200
NaCl (%)	11.46	6.92	39.61	
BOD_5_ (ppm)	3,050	982	67.80	≤100
COD (ppm)	7,132	1291	81.90	200

#### 3.2.10 Effectiveness of CA-GO as adsorbent

GO-based adsorbents are becoming more and more popular among researchers as adsorbents for eliminating pollutants from water sources. Table 6 is illustrated to assess capacity of CA-GO with other adsorbents for the elimination of Cr^3+^, Cu^2+^, and Cd^2+^. Notably, CA-GO showed reasonable metal absorption than the majority of other adsorbents.

**Table 6:** Comparison of reported adsorbent for Cr(III), Cu(II) and Cd(II) in literature with the present study.

Name of adsorbent	pH	Metal ion	q_max_ (mg/g)	Reference
GO aerogel	6.3	Cu^2+^	19.70	Mi et al. (2012)
GO-PAMAM	5.6	Cu^2+^	38.40	Yuan et al. (2013)
CMC-GO	5.5	Cd^2+^	134.04	Shakil et al. (2024)
Acrylic acid-grafted-sawdust (SD-g-AAc)	4.5	Cr^3+^	21.55	Ahmed et al. (2024)
MnFe_2_O_3_@TiO_2_-rGO	5.7	Cu^2+^	118.45	Chang et al. (2021)
GO/alginate hydrogel membrane	6.0	Cr^3+^	118.60	Bai et al. (2020)
ZnO nanoparticles	6.0	Cd^2+^	71.50	Kataria and Garg (2018)
Magnetic GO/MgAl-layered double hydroxide	5–6	Cu^2+^ Cd^2+^	45.05 23.04	Huang et al. (2018)
rGO-PDTC/Fe_3_O_4_	5.0 6.0	Cu^2+^ Cd^2+^	113.64 116.28	Fu and Huang (2018)
GO	4.0	Cr^3+^	366.30	Ahmed et al. (2021)
	6.0 7.0	Cu^2+^ Cd^2+^	193.05 231.45	Ahmed et al. (2023)
CA-GO	4.5 6.0 7.0	Cr^3+^ Cu^2+^ Cd^2+^	90.58 108.57 134.77	Present study

## 4 Conclusions

The ratio of GO to sodium alginate (10:1) and 6 % CaCl_2_ w/w solution can be used to make the CA-GO composite beads, which also yield good results for the removal of metals from aqueous solutions and tannery effluents. For Cr(III) at pH 4.5, 108.57 mg/g for Cu(II) at pH 6.0, and 134.77 mg/g for Cd(II) at pH 7.0, the adsorption capacity of CA-GO was measured. The utilized CA-GO composite was generated and utilized multiple times. For metals, the pseudo-second-order kinetic model produced a stronger correlation, and the CA-GO adsorbent's adsorption isotherm for Cr^3+^, Cu^2+^, and Cd^2+^ followed the Freundlich and Langmuir models. At varying temperatures, the enthalpy change and Gibb's free energy values for the CA-GO composite bead adsorbents were negative, indicating that the adsorption processes were physico-chemical and spontaneous. Other heavy metals can also be removed with this composite. Currently, graphene-based adsorbents are only used in lab settings to remove heavy metals. Therefore, in order to scale up for a large-scale practical application, more study is needed.

## CRediT authorship contribution statement

**Sobur Ahmed**: Conceptualization; Methodology; Visualization; Investigation; Writing - original draft; Writing - review & editing. **Tasrina Rabia Choudhury**: Resources; Validation; Writing - review & editing. **Md. Zahangir Alam**: Supervision; Funding acquisition; Writing - review & editing; Validation. **Mohammad Nurnabi**: Conceptualization; Project administration; Supervision; Writing - review & editing; Validation.

## Declaration of competing interest

The authors declare that they have no known competing financial interests or personal relationships that could have appeared to influence the work reported in this paper.

## Data Availability

No data was used for the research described in the article.
